# ‘Personalised evidence’ for personalised healthcare: integration of a clinical librarian into mental health services – a feasibility study

**DOI:** 10.1192/pb.bp.112.042382

**Published:** 2014-02

**Authors:** Rachel Steele, Paul A. Tiffin

**Affiliations:** 1 Lanchester Road Hospital, Durham; 2 Wolfson Research Institute for Health and Wellbeing, Stockton on Tees

## Abstract

**Aims and method** To evaluate the feasibility of integrating a clinical librarian (CL) within four mental health teams. A CL was attached to three clinical teams and the Trustwide Psychology Research and Clinical Governance Structure for 12 months. Requests for evidence syntheses were recorded. The perceived impact of individual evidence summaries on staff activities was evaluated using a brief online questionnaire.

**Results** Overall, 82 requests for evidence summaries were received: 50% related to evidence for individual patient care, 23% to generic clinical issues and 27% were on management/corporate topics. In the questionnaires 105 participants indicated that the most common impact on their practice was advice given to colleagues (51 respondents), closely followed by the evidence summaries stimulating new ideas for patient care or treatment (50 respondents).

**Clinical implications** The integration of a CL into clinical and corporate teams is feasible and perceived as having an impact on staff activities. A CL may be able to collate ‘personalised evidence’ which may enhance individualised healthcare. In some cases the usual concept of a hierarchy of evidence may not easily apply, with case reports providing guidance which may be more applicable than population-based studies.

The challenges of translating research into clinical and managerial practice have long been recognised.^1–3^ In principle, psychiatrists are amenable to evidence-based practice^4^ but despite this, research is inconsistently implemented into mental healthcare. One study^5^ reported that 65% of primary (mainly pharmacological) psychiatric in-patient interventions were based on ‘high-level’ evidence. However, another study reported^6^ that more than 50% of primary care and community patients with depression do not receive adequate antidepressant dosages and less than 10% receive evidence-based psychotherapy. There is also evidence that psychiatry trainees who are aware of research findings that question the relative effectiveness of newer antipsychotics were more likely to prescribe them than those who were unaware of the research.^7^

‘Passive’ knowledge translation (the synthesis and dissemination of research) is generally ineffective,^1–3^ thus the publication of journal articles alone is unlikely to result in behaviour change.^8^

Clinical librarians (CLs) have received little attention in knowledge translation. They provide ‘information to... support clinical decision making’^9^ and management/service delivery. It has been proposed^10^ that librarians should be transformed into ‘informationists’, meeting clinicians' need for synthesised evidence, as ‘There is no robust system to identify the information needs of today's psychiatrists in the UK’.^11^ However, there is one existing report on the potential benefits of embedding evidence in practice in community mental health teams.^12^ For this reason we evaluated the feasibility of routinely utilising a CL in order to facilitate knowledge exchange in a mental health setting. This intervention aimed to investigate whether evidence summaries produced by a multidisciplinary team integrated CL could meet a perceived need in a mental health trust and to report what enquiries would be raised.

## Method

Three clinical teams (one in-patient, two community) and the Trustwide Psychology Research and Clinical Governance Structure were recruited as initial pilot sites within Tees, Esk and Wear Valleys NHS Foundation Trust. This is a large trust which provides mental health and learning disability services to a region within north-east England. The CL aimed to embed knowledge of clinical evidence by attending clinical forums/consultations or supervisions/continuing professional development sessions to produce evidence summaries specific to individual patients, wider clinical problems or managerial work streams. To support the Psychology Research Structure, the CL completed literature reviews relating to organisational issues and provided training for psychology research assistants.

The approach used to produce the evidence summaries was the rapid review, which uses systematic review methods to search for and appraise research in a shorter time frame than would be needed to produce a systematic review.^13^ All requests for evidence summaries were recorded by the CL and classified according to the broad category of request (i.e. relating to individual clinical patients, generic issues of concern to the whole multidisciplinary team or clinical service, or management and corporate topics). Questions which could be classified as representing one of the four ‘classic’ types of clinical question (aetiology, diagnosis, prognosis or treatment) were also recorded as such by the CL.

Individual evidence summaries were evaluated via an online questionnaire. In the questionnaire, participants were first asked for basic details (their professional group and work base). They were then questioned on their views of the relevance, clarity and quality of evidence summaries via a set of subjective graded response options. Each graded response option question had five relevant levels; for example, for the question about clarity of the evidence summaries, options were from 1 (very unclear) to 5 (very clear). Participants were asked to indicate (via a set of closed question alternatives) whether they would have tried to find the information synthesised in the evidence summary themselves if the pilot CL service had not been available. They were also asked whether the evidence summary mainly confirmed their existing ideas, or whether it stimulated new ideas (or both). An important question asked participants to

**Fig 1:**
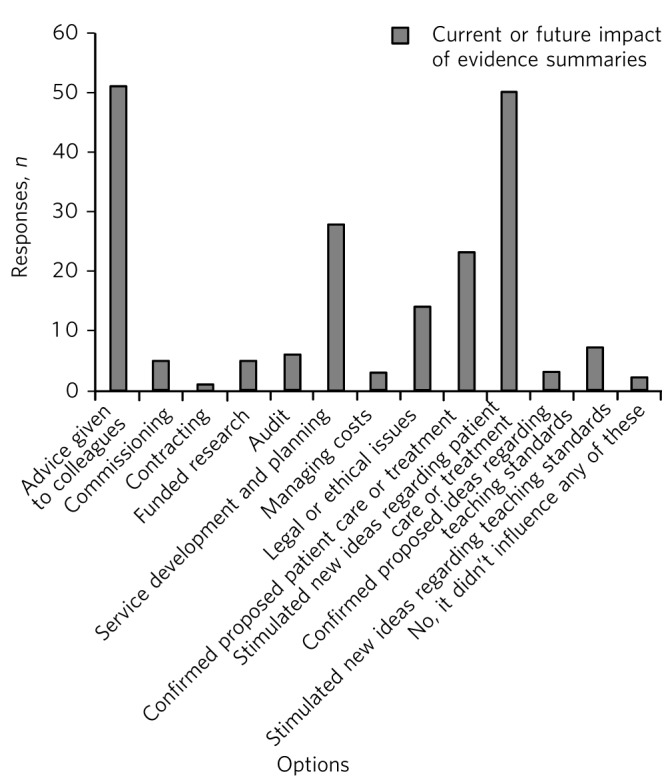
Trust staff's responses on (actual or anticipated) impact of evidence summaries.

indicate as many options as they felt were appropriate in terms of impact or potential impact of the evidence summaries. This set of drop-down options was taken from the NHS Library and Knowledge Services (England) and the Strategic Health Authority Library Leads (SHALL) impact toolkit (www.libraryservices.nhs.uk/forlibrarystaff/impactassessment/impact_toolkit.html). The questionnaire also contained a set of open questions in which participants were asked to assess the likely impact on their practice/patient care of not receiving the information synthesised in the evidence summary; and if possible to cite an example where the evidence summary had either changed previous views or where it had confirmed what they already knew, together with any further comments. The CL adapted two existing evaluation questionnaires to create a purpose-specific instrument, following input from the then library and information services manager. The questionnaire was delivered via Survey Monkey website (www.surveymonkey.com).

## Results

During the 1-year intervention period the CL received 82 requests for evidence summaries, in three broad categories of request. The first category related to the classic evidence-based practice conception of evidence being applied by the individual clinician to the individual patient (50% of summaries); the second to generic issues of concern to the whole multidisciplinary team/clinical service (23% of summaries); the third was for evidence summaries to be completed on management/strategic/corporate topics (27% of summaries).

Fifty-seven evidence summary topics broadly represented one of the four ‘classic’ types of clinical question: aetiology (26%), diagnosis (5%), prognosis (9%) or treatment (60%). The most common type of clinical question was for evidence relating to treatments, but the large number of requests relating to other information needs, particularly aetiology, is novel.

### Questionnaire results

At the end of the pilot period (September 2012), 105 responses to the online questionnaire had been received (Fig. 1).

### Case studies of impact on practice

The impact of the evidence summaries cannot be easily quantified because the trajectory from research into practice is not always direct and immediate in a complex field such as mental health. Some evidence summaries may have more subtle or long-term impact; for example, when a consultant psychiatrist was asked whether their views had changed following receipt of an evidence summary on clients with intellectual disabilities being carers for parents, they indicated that, prior to receipt, they would have been ‘more inclined to advocate moves to separate accommodation for people with [intellectual disability] and aged parents’.

Nevertheless, some striking examples of demonstrable effects on practice have emerged.

#### Evidence request 1: What is the evidence base for using electroconvulsive therapy (ECT) to treat the positive symptoms of schizophrenia in a patient with comorbid Parkinson's disease?

Request 1 provides an example where the timely provision of information prevented delays in the initiation of appropriate treatment. In addition to influencing current treatment it can be seen that the evidence summary was also anticipated to have an effect on future treatment.

Response from consultant psychiatrist, without receiving the CL evidence summary:

‘It is likely [that] the proposed treatment [would either have been delayed] until I had time to complete the review myself or we would have gone ahead without the clinical confidence that the summary has provided.’

‘I have never given ECT to someone with schizophrenia so it informed of the evidence in this area. I will now consider it as a potential option for treatment refractory clients in the future, particularly if they have neurological comorbidities.’

See Fig. 2 for an outline of the level of evidence utilised to answer this question.

#### Evidence request 2: What are the (psychiatric) effects of butane gas misuse and what symptoms might accompany withdrawal?

This request shows an example of clinical staff encountering an uncommon clinical problem and requiring information to ascertain the effects of the butane gas misuse on a patient's psychiatric presentation.

Response from consultant psychiatrist:

‘I think this one could be a good example of an “immediate response” to a difficult clinical question... [withdrawal from butane gas misuse] was something we had theorised about but had no knowledge of.’

#### Evidence request 3: What does the research literature tell us about creativity and mental illness (specifically bipolar disorder)? Is there any evidence to guide prescribing decisions to preserve a patient's creativity?

Request 3 shows an example of where a clinician's need for research to inform prescribing practice was clearly personalised and could not solely be met by sources such as the National Institute of Health and Care Excellence (NICE) guidelines, which provide general answers in the form of general guidelines.

**Fig 2:**
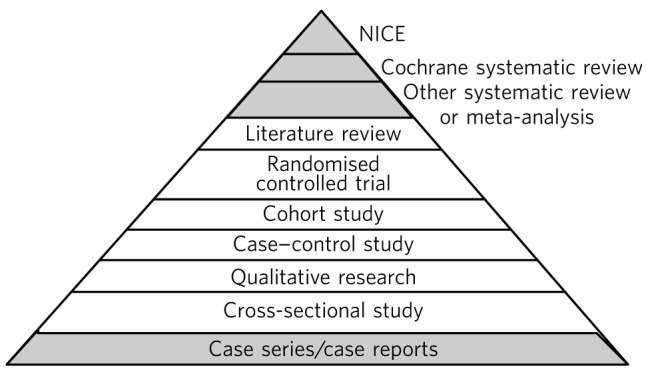
Research design pyramid, with highlighted levels indicating the designs which featured in studies synthesised by the clinical librarian to provide an answer to evidence request 1 (individual patient).

Response from consultant psychiatrist, without receiving the CL evidence summary:

‘I would likely have tried a NICE-recommended medication that was not recommended for this patient's unique presentation.’

#### Evidence request 4: What may be the influence of the menstrual cycle on psychiatric presentations?

This question was generated during a team discussion. Team members had previously hypothesised about the possible influence of menstruation on psychiatric presentations. The evidence summary has stimulated the multidisciplinary team to make changes in their clinical care, as the modern matron's response shows.

‘The clinical ward team has used the evidence summary to influence care by producing action plans to implement the findings, e.g. ascertaining last menstrual period... on admission and encouraging patients to use a menstrual cycle diary.’

#### Evidence request 5: Provide evidence summary on the topic of intrinsic motivation, mistake proofing, self-inspection and commitment in health professionals.

Request 5 highlights an instance where an evidence summary informed complex managerial/organisational development issues with implications for a number of areas, including patient safety. A manager's response to the summary shows how the themes from the research literature highlighted by the CL changed previous ideas they had on the topic:

‘Feedback is probably more effective when baseline performance is low, the person giving the feedback is a supervisor or colleague, when feedback is given in more than one format, and when it includes explicit targets and an action plan.’

#### Evidence request 6: How can the evidence-base illuminate ‘boredom’ among psychiatric in-patients?

Request 6 is a potent example which clearly demonstrates how research ‘can illuminate psychiatric practice in a more holistic way than purely by applying “scientific” evidence in the practice of “biological” psychiatry’.^14^ Figure 3 shows that, although NICE guidelines and randomised controlled trials (RCTs) were available to illuminate boredom among psychiatric in-patients, other research designs which are conventionally considered to be ‘lower’ in the evidence hierarchy were also synthesised by the CL.

Responses from a consultant clinical psychologist and occupational therapist show that the topic of boredom among in-patients has clear global importance for mental health services.

Consultant clinical psychologist: ‘The “boredom” in acute in-patients literature review has been extremely valuable to a variety of services across the trust including learning disability, forensic and older adult in-patient services. This has allowed these services to evaluate their current ward environments against the evidence and recommendations within the review. In my role as lead consultant clinical psychologist for research and clinical governance I have disseminated this review widely and the response has been extremely positive.’

Occupational therapist: ‘This paper offered a thorough understanding of a topic which is identified as a problem at every

**Fig 3:**
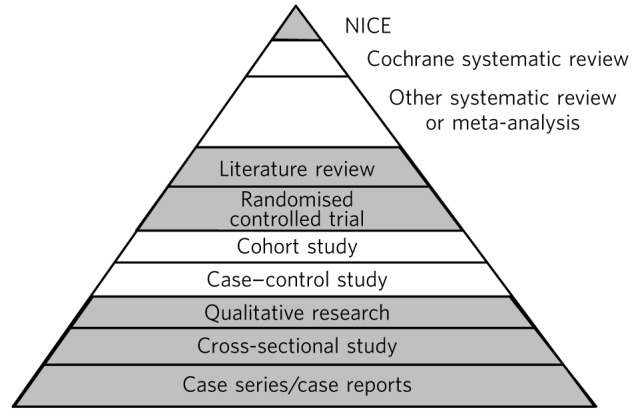
Research design pyramid, with highlighted levels indicating the designs which featured in studies synthesised by the clinical librarian to provide an answer to evidence request 6 (broader clinical).

Care Quality Commission commissioners' visit and clearly indicates a direction for in-patient services.’

Demonstrable influence on the practice of a clinical ward team is also highlighted as the boredom evidence summary has resulted in the following developments in the clinical ward team which received the CL evidence summary:^15^

- a morning handover between the nursing and intensive support teams making use of the patient's workbook so that intensive support staff are better briefed as to the activities/interventions likely to be most appropriate for the individual patient on that day
- intensive support team assessing the uptake of activities and noting when an activity has been offered, but is not taken up, by the patient
- since early 2012, multidisciplinary team meetings have been signposted to specific times each day to free up the nursing team for more one-to-one interactions with patients.

### Overall responses to Clinical Librarian Project

One respondent, a consultant psychiatrist, considered the contribution of the CL within the wider context of Trust responses to clinical governance, commenting: ‘When compared with all the other aspects of clinical governance the [multidisciplinary team] practises, I have no doubt that the addition of the clinical librarian to the team has made the single largest improvement’.^14^ A consultant clinical psychologist likewise perceived the CL role in the context of clinical governance and as integral to supporting research and development and issues core to the business of mental health trusts, commenting:

‘The clinical librarian role has been an extremely helpful resource in supporting the development of the psychology research and clinical governance framework and incredibly helpful in terms of supporting the national and regional quality and outcomes work relating to the payment by results agenda. I would fully support the continuation of this role and regard it as a critical component of the psychology research and clinical governance framework’.

## Discussion

This CL intervention demonstrated that there is perceived value in the local production of ‘evidence summaries’ to address practitioners' information needs. Interest in the CL role spread beyond the pilot areas and requests for summaries were received from Trust senior management and other corporate services. Our findings suggest that a CL can be an effective knowledge broker, facilitating the interaction between producers and users of research.^16^ The nature of the requests for evidence was also illuminating; the large number of requests relating to information needs other than treatment strategies (e.g. aetiology) is a novel observation. This contrasts with findings from a previous study^17^ that the number of treatment-related questions outweighed those relating to other clinical areas (e.g. diagnosis). Additionally, a previous systematic review^18^ highlighted how previous discussions of the CL role have concentrated on clinician decision-making and patient care. However, the diversity of the information requests received in the present project suggests that the role of the CL extends beyond providing clinically oriented evidence summaries.

Our observation that it was predominantly senior staff who initiated the majority of requests (only two came from trainee health professionals) contrasts with a previous study that reported that the perceived need for evidence is primarily to be found among trainee clinicians.^19^ Thus, we would speculate, it is senior staff that may most benefit from the role of the CL in this context. The primary reason why the clearest benefits from evidence summaries may be realised by senior staff is likely to be because the summaries were written on ‘foreground’ rather than ‘background’ topics/clinical questions. The distinction between background and foreground clinical questions is clearly elucidated in the literature.^20,21^ Background questions tend to be posed by students or trainee clinicians. They are about well-established facts/general knowledge and are usually answerable by textbooks or electronic ‘point of care’ summaries such as Clinical Evidence, DynaMed or UpToDate. In contrast, foreground questions require a synthesis of highly targeted evidence on treatment, diagnosis, prognosis or aetiology. Foreground questions are best answered by the research literature rather than by textbooks because they require current evidence (textbooks may be out of date) and they are often so specific that they will not be covered in general reference works. It is therefore usually more complex and time-consuming to search for an answer to a foreground, than a background, question.

Although it is appropriate that ‘academic institutions and medical professional associations should contribute to collective efforts to summarise medical evidence and build... repositories of knowledge on the Internet’,^22^ this alone is unlikely to influence practice. The present project has provided a suggestion as to how CLs may produce evidence syntheses which meet demonstrable information needs locally and individually. Our experience also challenges the traditional evidence-based practice paradigm. This model suggests provision of training, enabling clinicians to formulate clinical questions, search the literature, and appraise and apply the evidence. Clinicians are likely to benefit from education in literature reviewing, and use of secondary sources may be an especially efficient way of following evidence-based practice.^23^ Nevertheless, there is evidence to suggest that relying on the ‘knowledge users’ to find and implement research findings is problematic.^24^ Thus, alternatives such as the production of intermediate evidence syntheses may support both clinicians and managers in becoming competent ‘evidence users’.^25^ Training of end users must thus increasingly encompass an understanding of the role of CLs as evidence facilitators.^26^ Our present findings also highlight a potential reason why some evidence-based practice educational interventions have not produced substantive behaviour change.

### Personalised evidence

The nature of the requests processed by the study CL suggests that the nature of ‘evidence’ perceived as required by knowledge users is more complex and subtle than proponents of evidence-based practice may assume. An evidence summary on ‘boredom’ in in-patient mental health settings is particularly instructive because it offers a challenge to perceptions by some practitioners that ‘evidence’ is of limited use, because ‘Most of psychiatry lies in a “grey zone” of clinical practice and may thus lie outside the scope of [evidence-based practice]’.^19^ These perceptions coincide with a mistaken view that evidence is synonymous with the RCT and therefore most applicable to ‘biological’ psychiatry.^27,28^ The fact that conventional ‘high-level’ evidence was available to illuminate issues relating to boredom (Fig. 3) may challenge perceptions that such evidence is not available to enlighten ‘greyer’ areas of psychiatry. Moreover, a multiplicity of research designs (not

**Fig 4:**
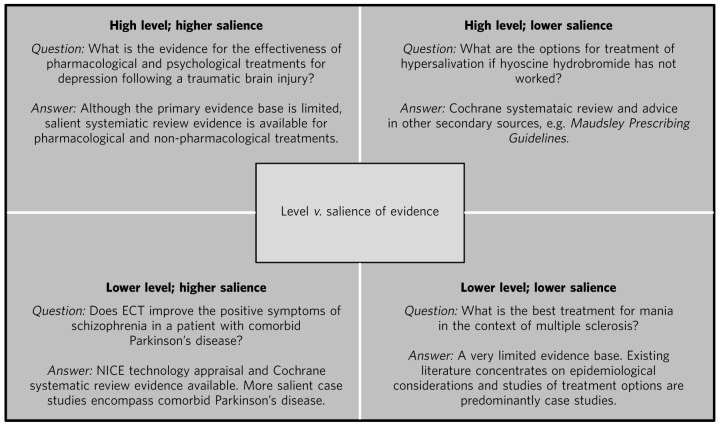
Matrix diagram showing tension between ‘salience’ (applicability to unique clinical problem) and ‘level’ (type of study design and methodological robustness) in using research evidence. ECT, electroconvulsive therapy; NICE, National Institute for Health and Care Excellence.

just RCTs) can make a valid contribution to a ‘grey’ topic and lead to changes in practice. Indeed, at times, clinical utility or relevance may work in the opposite direction to the ‘pyramid of evidence’, with case studies being more influential than wider-scale evidence. For example, consider the impact of the case of Victoria Climbié on children's Social Services^29^ or the effect of the killing of Jonathan Zito by Christopher Clunes on community mental healthcare.^30^ In such instances, the salience of research may apply in a different way to that envisaged by proponents of the hierarchical ‘evidence pyramid’ when research is required for particular circumstances (Fig. 2), with more stress placed on rich, detailed case reports rather than more generic, ‘higher-level’ evidence.

We would thus suggest a novel concept of personalised evidence. This concept relates to the process of searching for, and synthesising, evidence that provides the best fit to the clinical or organisational question at hand while drawing on (and weighting) evidence from the highest levels available. Thus, it acknowledges a tension between two dimensions: salience (the extent to which the evidence is specific to the problem of focus) and level (the level in the hierarchy of evidence from which the information is drawn). (See Fig. 4 for some illustrative evidence queries to show the tension between ‘level’ and ‘salience’ of evidence; see also Fig. 2 which highlights how case study research was synthesised by the CL, in addition to ‘higher-level’ evidence.)

### Personalised medicine

We would view the concept of personalised evidence as complementing the evolving concept of personalised medicine. To date, the main focus of this movement has been to utilise information on individual differences (notably genetics) to predict response to pharmacological treatment. Such ideas have been applied to mental health to some extent.^31–33^ However, it is inevitable that personalised medicine will need to enlarge its scope as differences in health-related behaviours (e.g. medication concordance) are likely to predict treatment response to a greater extent than pharmacogenetic factors (i.e. your hepatic enzyme expression is not relevant if you are not taking the tablets). Thus, we would promote a potential integration between the evidence-based practice paradigm and the personalised medicine paradigm with a widening in scope, taking account of patients' preferences and values – a central component of evidence-based practice.^34^

### Personalised research

As well as evidence being personalised to patients' clinical presentations, needs and values, research can also be personalised to the needs of the clinician so that it can have an impact on the patient-clinician dyad. Arguably, clinicians' information needs are inherently ‘personal’ and require a personalised response from a CL. For example, a consultant psychiatrist and mental health nurse may both require evidence to care for the same patient but a CL can select information appropriate to each, even if their questions are expressed in a similar manner. Clinicians' need for personalised evidence not only relates to their professional group but many highly individual factors such as the clinician's level of experience in their role, whether or not they have previously encountered the clinical problem, their current theoretical and practical knowledge and their interest in, and skills and expertise towards, using research evidence. Evidence can therefore be selected and synthesised by a CL so that the research can be personalised to the patient, personalised to the clinician, and consequently personalised to the unique patient-clinician encounter.

It should be highlighted that the reliance on ‘lower’ levels of evidence to guide actions does not sit well with methodologists from a frequentist tradition (which has dominated medical research culture), where the emphasis is on deriving certainty from establishing the unlikelihood of an event occurring through chance alone (i.e. *P*<0.05). In contrast, Bayesians view even subjective information (e.g. expert opinion, clinical experience) as informative and such approaches have been widely implemented in, for example, business, where often high-stakes decisions must be made under uncertainty.^35^ Moreover, the Bayesian approach utilises prior knowledge, where available, to increase our confidence in making inferences from newly observed data.^36^ In theory, this means that lower levels of evidence can be combined with emerging higher levels of evidence to increase our certainty about the probable outcome of a course of action.

### Limitations

Limitations in our pilot study must be acknowledged. The participants constituted a non-probability sample in that users of the service self-selected, therefore respondent bias may make our findings difficult to generalise to a wider setting. Second, we did not record the intentions of participants before they received the evidence syntheses so the retrospective nature of the study makes it potentially susceptible to recall bias. Although anecdotally the evidence summaries changed the course of some actions, it is not clear whether requests were generally an effort to seek confirmation and support for decisions rather than guidance. Last, this feasibility study focused on the perceptions of end users and did not record objective information on any wider scale changes in practice (e.g. concordance with NICE guidelines). Therefore future research should adopt a more robust prospective design, incorporating objective, as well as subjective, metrics of change and ideally also include data relating to patient experience. Future studies should also adopt a clustered (multilevel) design to account for possible differences between teams and services.
